# Stenting the ductus arteriosus: Case selection, technique and possible complications

**DOI:** 10.4103/0974-2069.41054

**Published:** 2008

**Authors:** Mazeni Alwi

**Affiliations:** Department of Paediatric Cardiology, Institut Jantung Negara (National Heart Institute), Jalan Tun Razak, Kuala Lumpur, Malaysia

**Keywords:** Cyanotic heart disease, palliation, ductal stenting

## Abstract

Ductal stenting is an attractive alternative to conventional shunt surgery in duct dependent congenital heart disease as it avoids thoracotomy and its related problems. With today's generation of coronary stents which have better profile, flexibility and trackability, ductal stenting may be achieved safely and with considerably less difficulty than previously described.

As in Blalock-Taussig (BT) shunt, ductal stenting is indicated mainly in duct-dependent cyanotic lesions chiefly in the neonatal period. Unlike the Patent ductus arteriosus (PDA) as an isolated lesion, the ductus in cyanotic heart disease has a remarkable morphologic variability. The ductus tends to arise more proximally under the aortic arch, giving rise to a vertical ductus or occasionally it may arise from the subclavian artery. It also tends to be long and sometimes very tortuous, rendering stent implantation technically impossible. The ductus in these patients may also insert onto one of the branch pulmonary arteries with some stenosis at the site of insertion. The ductus in Tetralogy of Fallot with pulmonary atresia (TOF-PA) tend to exhibit these morphologic features and to a lesser degree in transposition of great arteries with ventricular septal defect and pulmonary atresia (TGA-VSD-PA) and the more complex forms of univentricular hearts. In the preliminary angiographic evaluation, it is important to delineate these morphologic features as the basis for case selection.

Ductal stenting may be done by the retrograde femoral artery route or the antegrade transvenous route depending on the ductus morphology and the underlying cardiac lesion. The detailed techniques and essential hardware are described. Finally, major potential complications of the procedure are described. Acute stent thrombosis is the most serious and potentially catastrophic. Emergent treatment with thrombolytic therapy and mechanical disruption of thrombus are required. With proper case selection, appropriate technique and the right hardware ductal stenting provides reasonable short-medium term palliation in duct-dependent cyanotic heart disease.

## INTRODUCTION

Advances in surgical technique and post-operative care in the last two decades have led to improved survival and long-term outcome in children with complex cyanotic heart disease. Equally noteworthy, corrective surgery is increasingly performed at an earlier age and a lower body weight, obviating the need for palliative procedures in lesion-like Tetralogy of Fallot (TOF) in the more established centers. However, palliative Blalock Taussig (BT) shunt is commonly performed today in relatively complex duct-dependant lesions. Many of these conditions require staged palliation or can only be corrected at a later age. Hearts with single-ventricle physiology with pulmonary atresia and TOF with pulmonary atresia (TOF-PA) are examples of such conditions. Thus, in the modern era, BT shunt as a palliation is an operation that is almost exclusively performed in the neonatal period or early infancy.

Ductus arteriosus stenting, although not widely accepted, is nevertheless attractive as a less invasive alternative for first-stage palliation.[1–4] The focus of this article is the practical aspect of ductal stenting in the catheterization laboratory, providing a detailed step-by-step description of the procedure and of the equipments used with the objective of guiding paediatric cardiologists less familiar with this technique. A brief discussion on angiographic anatomy is also included to guide case selection. Potential complications and their possible prevention is also outlined.

## PREPARATION

Most of these patients are neonates or very young infants in whom the ducts are maintained open by PGE1 infusion. Our preference is to turn off the infusion the night before the procedure. The purpose of this is to have a reasonably constricted ductus arteriosus at the time of the procedure as ducts that are large (≥2.5 mm) will not be suitable for stenting. There would be a high risk of stent migration in such large ducts. However, a small number of patients cannot tolerate even a few hours without PGE1 infusion. In such cases, the PGE1 may be turned off just before or at the beginning of the procedure.

We prefer that the procedure is done under general anaesthesia and special attention be given to minimize hypothermia. Bowls of saline for flushing and contrast media should be kept in a basin of warm water throughout. It is highly recommended that an arterial line and a central venous line be secured after induction of anaesthesia for monitoring and administration of drugs during and after the procedure.

For the procedure itself, one femoral artery is cannulated with a 4-F sheath and one vein is accessed with 5-6 F sheath. Heparin 50 units/kg is given at the start of the procedure and repeated every 1-1½ hour. Baseline measurements of arterial blood pressure, central venous pressure (CVP) and blood gases are done.

Extrapolating from adult practice in coronary stenting, starting antiplatelet agent(s) a day before the procedure may be a worthwhile measure to prevent acute thrombosis, but convincing data would not be forthcoming given the low numbers of this procedure.

## PRELIMINARY ANGIOGRAPHIC STUDY AND CASE SELECTION

**Figure 1:**
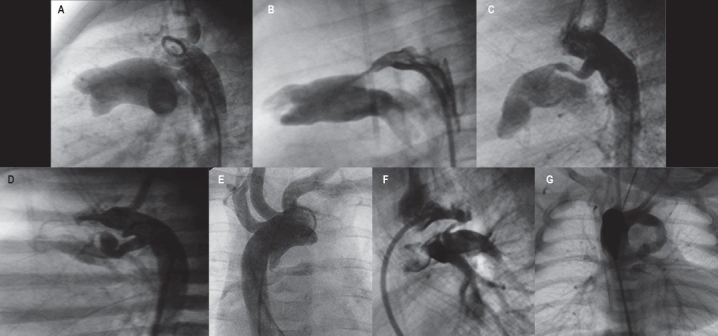
(A) Commonest form of PDA as an isolated lesion (Krichenko Type A) seen on the lateral projection. Cone-shaped ductus with wide ampulla and constriction at the pulmonary end arising from the proximal part of the descending aorta. (B-G) Variation of ductal morphology in duct-dependant cyanotic CHD. (B) Ductus in a neonate with tricuspid atresia resembling Krichenko type A PDA. (C and D) Ductuses in cyanotic CHD tend to arise more proximally, have a more elongated and tubular shape one or more curves. (E and F) Very proximal origin of ductuses, arising from the underside of the aortic arch are seen more commonly in TOF-PA. (G) A long tubular ductus arising from the subclavian/innominate artery in a patient with TOF-PA and right aortic arch.

**Figure 2:**
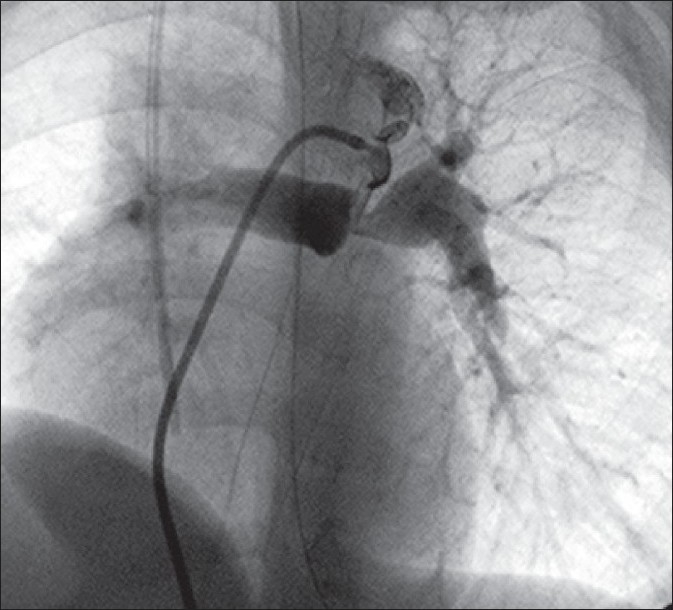
Ductus arteriosus in TOF-PA arising proximally from the underside of the aortic arch inserting onto the proximal part of the LPA. Significant stenosis of the LPA is present. The tip of a JR catheter passed transvenously into the aorta via the VSD is engaged in the ampulla for adequate visualization

In most of the patients, the diagnosis would have been established by echocardiography. The role of angiography is to have a detailed evaluation of the ductus arteriosus morphology. As opposed to isolated persistence of the ductus arteriosus, in duct-dependent cyanotic congenital heart disease (CHD), the ductus arteriosus is markedly varied with regards to its origin from the aorta, size and shape, length, tortuosity and its insertion onto the pulmonary artery [Figure 1].[5] The typical duct in cyanotic CHD is elongated with one or more curves and arises more proximally from the underside of the aortic arch. It often becomes constricted as it inserts onto the pulmonary artery. Some ducts are unusually long and have a very tortuous course with one or more constrictions. In such cases, ductal stenting may neither be technically feasible nor safe. The ductus arteriosus in tricuspid atresia or pulmonary atresia with intact ventricular septum (PAIVS) has a more normal origin from the proximal descending aorta and a more normal short straight course with a constriction as it inserts onto the top of the main pulmonary artery (MPA). These are ducts that are most suitable for stenting, and the procedure can be expected to be relatively uncomplicated with little risk of late left pulmonary artery (LPA) stenosis. On the other hand, in other forms of cyanotic CHDs, especially TOF-PA, the ductus arteriosus morphology is often more complex, i.e., long, tortuous, and arise more proximally from the underside of the aorta. They have a tendency to insert onto the proximal LPA (in left aortic arch), causing a significant constriction and “kinking” of LPA [Figure 2]. Ductus arteriosus stenting in such cases is likely to accelerate branch pulmonary artery stenosis and the procedure is likely to be more challenging. After our initial experience, we feel such ductus arteriosus morphology is a relative contraindication to stenting. In hearts with single-ventricle physiology destined for the Fontan track, this is perhaps an absolute contraindication, as an accelerated LPA stenosis is likely to undermine a successful outcome.

It is thus crucial that the initial angiographic study gives adequate information with regards to where the ductus arises from the aorta, its shape, course and tortuosity, its site of insertion onto the pulmonary artery, presence of branch pulmonary artery stenosis (stenoses), especially those that insert onto the LPA. The projections that are most helpful are the straight lateral and the “4-chamber” view (LAO 25-30°, cranial 25-30°) to open up the pulmonary artery bifurcation and show the proximal LPA stenosis if this is present (for Dextrocardia, it is the RAO equivalent).

From these angiograms, measurements of the narrowest part of the ductus (usually the distal part where it inserts onto the pulmonary artery), the size of the ampulla, the body of the ductus for the long tubular type and diameters of the branch pulmonary arteries are made.

As elaborated above, our exclusion criteria are ductuses that are not sufficiently constricted at the pulmonary end (≥2.5 mm) because of the risk of stent migration and those in whom there is pre-existing branch pulmonary artery stenosis at the site of ductal insertion, especially in hearts with single-ventricle physiology.

## DUCTAL STENTING TECHNIQUES

Our approach to ductal stenting is based largely on ductal morphology, particularly on how the ductus arises from the aorta. In the foregoing, we describe four broad techniques that would cover the vast majority of ductus arteriosus morphology, which are suitable for stenting. The group A ductus is the most amenable to stenting, the procedure generally being straightforward and the least likely to develop branch PA stenosis in the medium term. The first technique (A) is described in a detailed step-by-step manner including choice of guidewire, stent size and length, positioning and expansion of stent. Some aspects of this, such as repeated hand injections for accurate positioning, are also integral to the other techniques and they will not be repeated. The catheters, guidewires, balloon and stents that are deemed essential for the procedure are listed in Table 1.

**Table 1:** Equipment needed for ductal stenting

1	Diagnostic catheters
	4-F pigtail and 4-F Judkins Right for femoral artery route
	5-F JR for transvenous route
2	Catheters for stent delivery
	4-F long sheath (Cook Inc, Bloomington, IN, USA)
	5-F or 6-F Judkins Right, XB
	Judkins Left guiding catheters
	Tuohy Borst Y-connector
3	0.014″ guidewires Hi-Torque Balanced Middle Weight (BMW) Universal (Abbott Vascular, Santa Clara, CA, USA) Choice PT extra support (Boston Scientific Corporation, Natick, MA, USA) Stiff wires for acute stent thrombosis
	Hi-Torque Pilot 150 (Abbott Vascular, Santa Clara, CA, USA)
	Persuader (Medtronic Inc, Minneapolis, MN, USA)
4	Coronary stents 3.5 mm, 4.0 mm, and 4.5 mm
	Lengths 8-18 mm
	Coronary balloons 2.5-4.5 mm

### (A) Ductus arteriosus arising from the proximal descending aorta

**Figure 3:**
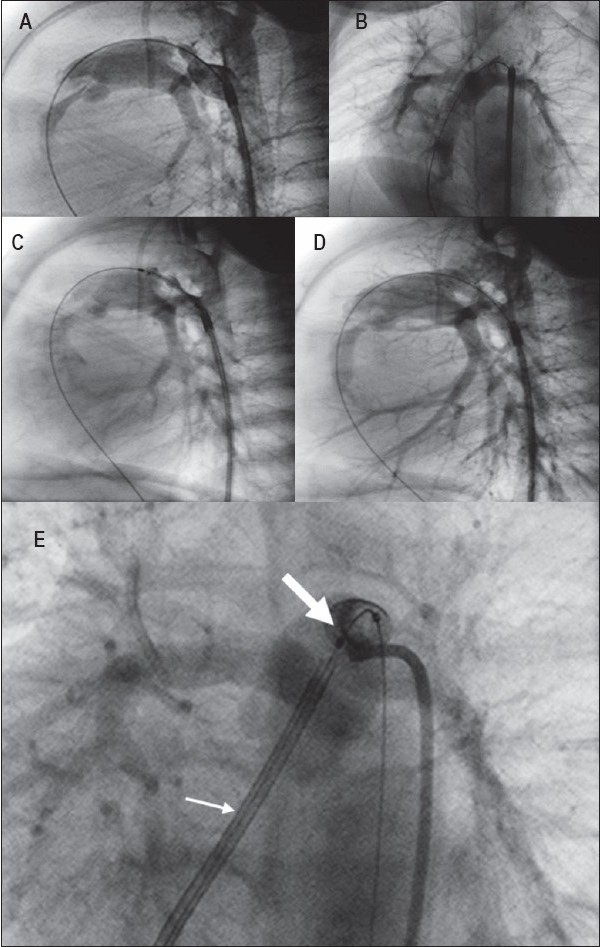
PA-IVS with moderate RV hypoplasia. Ductal stenting by retrograde femoral artery route after radiofrequency and valvotomy balloon dilatation. (A) Lateral projection shows - almost like Krichenko type A ductus but more elongated, with a very tight constriction at the pulmonary end. The tip of 4-F long sheath is positioned near the ampulla. 0.014” guidewire is passed through catheter across the ductus using a JR catheter to the RV, RA and IVC. (B) Angiogram in LAO 30° cranial 25° projection demonstrating the insertion of ductus onto the roof of MPA away from the LPA origin. (C) Balloon-stent ensemble positioned along the length of ductus ready for expansion. (D) Post-expansion angiogram. Entire length of ductus is covered by stent with no encroachment of origin of branch pulmonary artery. Ductal stenting by transvenous route in another patient with PA-IVS after RF valvotomy. (E) JR guiding catheter is used for stent delivery (small arrow). Expanded stent (big arrow) is seen well clear of LPA and RPA origins

These ductus arteriosus are seen more in PAIVS, critical pulmonary stenosis (PS) and tricuspid atresia. They often resemble the commonest form of isolated PDA, the Krichenko type A, i.e., having a wide ampulla and tapering down to a tight constriction.[6] It arises from the proximal descending aorta, has a relatively short, straight course anteriorly and superiorly to insert onto the dome of the MPA above the left and right pulmonary artery origins on the lateral projection. Hence, branch pulmonary artery stenosis is less likely to occur. Not uncommonly, the body may be more elongated and tubular but not tortuous [Figure 3A,B].

Once preliminary angiograms have been performed with a 4-F pigtail catheter, the short 4-F sheath is replaced with a 4-F long sheath (Cook's). The tip of the long sheath is placed just below the ductal ampulla. A 4-F Judkins' Right (JR) catheter is maneuvered into the ampulla and a hand injection is done under fluoroscopy to guide the crossing of ductus. A 0.014″ coronary wire is used to cross the ductus and enter into the pulmonary artery. It is important to have a Y-connector (Tuohy Borst) secured at the hub of the catheter to prevent blood loss as the JR has a 0.035″ lumen. Our choice of wire is the choice PT extra support (Boston Scientific) as it has a short, floppy hydrophilic tip for negotiating the constriction and distal tortuosity if present and a relatively stiff body to enable tracking of balloon-stent across the ductus. The floppy tip and a sufficient length of the stiff body should be anchored securely in a distal branch pulmonary artery. Repeat the angiograms using small volume of contrast with hand injection via the side-port of the long sheath, in the straight lateral and four-chamber projections for measurements of the ductus. The guidewire across the ductus tends to straighten it. A more accurate measurement of the length of the ductus for selection of stent length is made (In this type of ductus not much “straightening” occurs with placement of guidewire as in the more tortuous forms).

For patients weighing 3.0-4.0 kg, we choose a 4.0-mm diameter stent and for those weighing 4.0-5 kg, we would use a 4.5-mm diameter stent. For patients weighing <3.0 kg, we would choose a 3.5-mm diameter stent. Patients below 2.5 kg should be excluded as the 4-F long sheath may cause major damage to the femoral artery. The stent length chosen should ideally cover the entire ductus, expecting that 15-20% shortening will occur upon full expansion. We use pre-mounted stents in all cases.

To prevent displacement of stent from balloon during insertion of the balloon-stent ensemble into the long sheath, the distal tip of short 4-F sheath used previously for the pigtail angiograms is cut and used to protect the stent as it is being pushed across the rubber valve. As the stent is advanced towards the ampulla, it is necessary to ensure that the guidewire remains as straight and taut as possible. Once the tip of the stent is across the narrowest part of the ductus (the air column, endotracheal tube or central venous lines are useful landmarks), hand injections are repeated in the same projections and fine adjustments are made to position the stent accurately, such that enough stent length is in the main pulmonary artery and secondly there is no protrusion into the aorta at the other end. Stents protruding into the aorta may be difficult to re-cross at follow-up catheterization. Once satisfied with the positioning, the assistant inflates rapidly while the operator keeps the balloon catheter fixed and steady as the stent tends to jump forward during inflation (“melon-seeding”). Once fully expanded, the balloon is deflated and angiograms are repeated [Figure 3C,D]. It is important to look for branch pulmonary artery stenosis and ensure that both the pulmonary and the aortic ends of the ductus are covered. At this stage, with the wire still across, it is still relatively easy to implant another stent. It is also wise to keep the wire across the ductus for about 15 min in the rare/uncommon event of acute stent thrombosis. Finally, the guidewire and long sheath are removed. If the patient does not have another arterial line for blood pressure monitoring and blood gases, the patient returns to the ICU with the long sheath.

### (B) Ductus arteriosus arising from proximal or middle part of the aortic arch (“vertical” ductus arteriosus)

These ducts are most commonly seen in TOF-PA, as well as more complex forms of cyanotic heart disease such as TGA-VSD-PA or hearts with single-ventricle physiology. They arise from the underside of the arch opposite the origin of the left subclavian artery (LSCA) or its equivalent in a right aortic arch. The ductus tapers down vertically and often forms a kink as it inserts onto the pulmonary artery in a tight constriction. Quite commonly, it inserts onto the proximal part of the LPA in a left aortic arch [Figure 3]. Kinking and stenosis of the LPA may already be visible even in the neonatal period.

**Figure 4:**
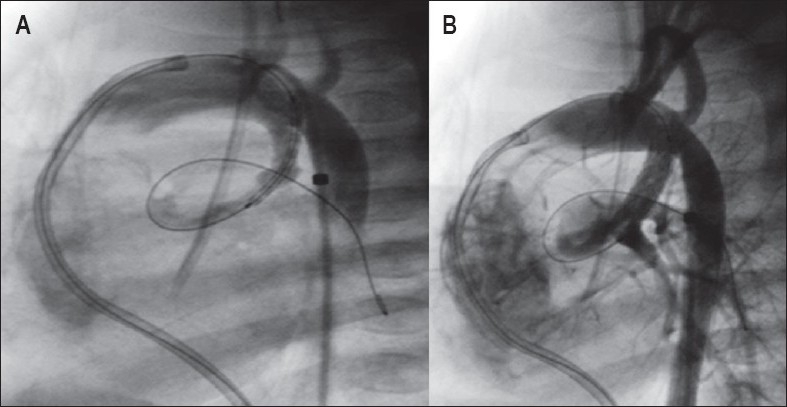
Lateral projection - ductus arteriosus in TOF-PA arising from the underside of the arch (vertical ductus), on the opposite side between the origins of the left common carotid and left subclavian artery. Ductal stenting by antegrade transvenous approach. 0.014” guidewire well-anchored in a distal branch pulmonary artery. Balloon and stent across the entire length of the ductus ready for expansion. The tip of JR guiding catheter has been kicked back out of the ductal ampulla with the passage of balloon stent (A). Expanded stent

This type of ‘vertical’ ductus arteriosus generally is not amenable to stenting via the retrograde, femoral artery route as it is very difficult to engage the ampulla, and even more so, securing a stable guidewire position for tracking of balloon-stent ensemble. Unless one is familiar with axillary artery cannulation or carotid artery cut-down, stenting of this type of ductus may be achieved by the transvenous route except in hearts with single-ventricle physiology [Figure 4]. A 5-F JR catheter can be used for the initial right heart catheterization and then placed in the ascending aorta via the VSD. Even a pigtail catheter may not produce good quality angiograms for detailed ductus and pulmonary artery morphology. This is best achieved by using a 6-F XB coronary guiding catheter with its tip cut off to form an “inverted J”. With an exchange length guidewire, the 5-F JR catheter in the aortic arch is replaced with the modified XB guiding. The tip is then maneuvered to engage the ductal ampulla and preliminary angiograms of the ductus and pulmonary arteries are taken in the same projections as in (A). The rest of the procedure such as guidewire anchoring, measurements, stent length and diameter choice, accurate positioning and expansion are as previously described. If LPA stenosis is seen, this should be monitored closely and one needs to be prepared to shunt the left side and incorporate pulmonary arterioplasty at the time of definitive repair. A note of caution is the possibility of complete heart block as the stiff guiding catheter rubs against the ventricular septal defect rim where the conduction bundle is located. It this persists, it may be wise to discontinue rather than temporarily pace the patient.

### (C) Intermediate origin

In this group, the ductus arteriosus arises more proximally than those in isolated persistent ductus arteriosus, but not as extreme as in group (B). They arise from the side opposite the origin of the LSCA; hence, a less vertical take-off than in group (B) ductus. These ducts tend to be long and tubular with a more tortuous course especially near its insertion onto the pulmonary artery [Figure 4]. In general, stenting this type of ductus is best approached by the femoral artery route as in (A). However, the most challenging part is crossing the ductus and anchoring it in the pulmonary artery for stable guidewire position.

As in (A), after the preliminary angiograms with a 4-F pigtail catheter, a 4-F long sheath is placed in the aorta with its tip near the ductal ampulla. However, instead of a 4-F JR catheter, a 4-F pigtail with its tip cut off to form an inverted J, is often required to engage the ductal ampulla and perform selective angiograms of the ductus and pulmonary arteries. Due to its tortuosity and tight constriction at its pulmonary end, crossing it with choice PT extra-support may be very difficult or not possible. The BMW wire (Abbott Vascular) or others with a similar degree of floppiness are preferred. The tip of the wire is gently advanced through the ductus while simultaneously twisting it clockwise and anti-clockwise with a torquer. This may require several attempts as the tortuosity may force the wire tip to spring out the ductus. Once a good length of the wire is in the pulmonary artery and securely anchored, the tortuosity tends to straighten out and the ductus takes a more horizontal orientation. Repeat hand-shot angiograms via the side-port of the long sheaths in the projections above will show this well and this should be the basis of measurements for choice of stent length. The rest of the procedure is as in (A).

**Figure 5:**
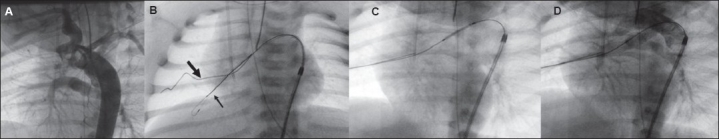
(A) LAO-cranial projection - ductus arteriosus in PA-IVS with severe hypoplasia of the RV. Proximal origin of ductus (opposite the origin of the left subclavian artery), but transvenous route is not feasible as there is no RV-PA continuity. (B) The tip of a cut pigtail is engaged in the ductal ampulla. A soft 0.014” guidewire (BMW) is first used to cross the ductus (small arrow), allowing some straightening of the ductal course. A stiffer wire, Choice PT extra support (big arrow) is then passed across for better tracking of balloon-stent. (C) Balloon-stent positioned along ductal length ready for expansion, softer BMW wire removed. (D) Fully expanded stent

Occasionally, the balloon-stent ensemble may not be able to track the softer BMW wire across the ductus. The balloon-stent should be taken out and a second, stiffer wire (Choice PT extra-support) may now be able to cross the straightened-out ductus. The BMW wire is then removed and the balloon-stent is tracked over the new wire, repeating the steps as in (A) above [Figure 5].

### (D) Ductus arteriosus arising from the subclavian artery

**Figure 6:**
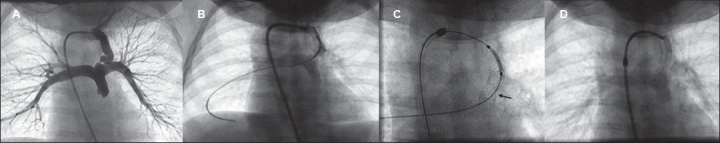
(A) AP projection - long tubular ductus arteriosus arising from the innominate/left subclavian artery with a sharp curve distally. Very tight constriction is seen at pulmonary insertion. Ductal stenting via femoral artery route. (B) 4-F long sheath is positioned in the innominate artery. 0.014” guidewire anchored in a distal pulmonary artery branch. The first stent is covering the distal two-thirds of the ductus and is ready for expansion. (C) First stent is expanded (arrow). Second shorter stent is covering the remainder of the ductus and is ready for expansion. (D) Both stents after expansion are seen covering almost the entire length of ductus. A single, long stent is likely have a great difficulty tracking over the wire and when fully expanded may cause distortion of the pulmonary artery

In this least common of ductus arteriosus, comprising less than 5%, the ductus has the peculiar appearance of a BT shunt. The long tubular ductus arises from the left subclavian artery (LSCA) (or from the right subclavian artery in a right aortic arch) and joins the pulmonary artery in a roughly perpendicular fashion. There is usually a tight constriction at the site of pulmonary insertion. Stenting this type of ductus can be approached by the retrograde trans-arterial method as described in (A) above. The tip of a 4-F long sheath is placed near the origin of the LSCA. A 4-F JR catheter is used to engage the proximal part of the LSCA, through which an extra-support choice PT wire is used to cross the ductus and anchored deeply in a branch pulmonary artery. As described above, measurements for selection of stent length are best made with angiograms after guidewire positioning. With long tubular duct, a single long stent is likely to have great difficulty in tracking the wire. It is best to place a short stent in the distal part of the ductus to cover just enough of the ductal constriction and a second stent for the proximal part [Figure 6].

## POTENTIAL COMPLICATIONS

### (A) Acute thrombosis

**Figure 7:**
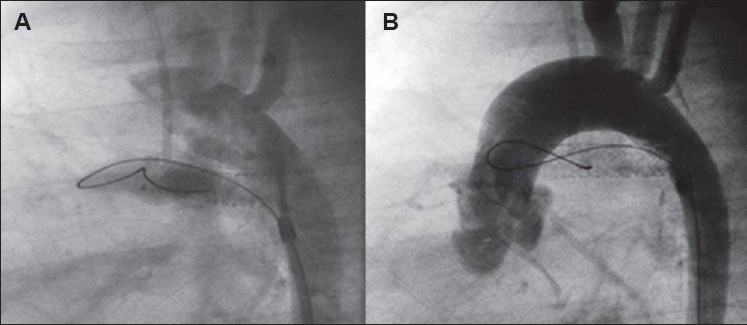
Acute stent thrombosis. (A) Immediate post-stent expansion is showing - good flow through the stented ductus. (B) Ten minutes post-expansion. The guidewire is still across the stent. There is rapidly falling oxygen saturation with very severe desaturation. The entire stent is filled with thrombus

This is not a common complication, perhaps a rate of 2-3% can be expected as more procedures are performed, but it is very serious and life threatening. It presents in a dramatic fashion and requires immediate action. Typically, the oxygen saturation reaches normal or near normal level immediately after stent expansion. In the few cases encountered, the oxygen saturation fell rapidly as preparations were made to wrap up the procedure. Angiography typically reveals thrombus occluding stent completely or partially [Figure 7]. If the guidewire is still across the ductus, a small balloon (2.5-3 mm) may be passed into the stent and inflated several times, advancing the balloon forward towards the pulmonary artery each time. The purpose of this maneuver is to mechanically break up the thrombus. Thrombolytic therapy with streptokinase or recombinant tissue plasminogen activator is also recommended at the same time and maintained for at least 24 h. However, there is a real risk of bleeding complications with thrombolytic therapy, especially if a surgical shunt is required when adequate oxygenation cannot be maintained. Acute thrombosis occurs fairly soon after stent expansion; it is thus advisable that the wire be kept across the ductus for about 15 min before terminating a successful procedure. If thrombosis occurs after removal of the wire, a stiff coronary wire normally used in coronary interventional work for chronic total occlusion, e.g., Pilot 150 (Abbott Vascular) or Persuader (Medtronic) may be employed to “drill” through the thrombus. A balloon is then tracked over the wire to mechanically break up the thrombus as above. Acute thrombosis occurs even in those patients who are adequately heparinized during the procedure. It is not known if giving anti-platelet agents the day before the procedure would reduce this complication, but it may be worthwhile extrapolating this adult coronary intervention practice to neonatal ductal stenting. If ductal stenting gains wider acceptance, this is an area that merits investigation.

### (B) Spasm of the ductal arteriosus

Fortunately, this is an uncommon complication (<1%) even in those ductuses that are severely constricted at the pulmonary end. It tends to occur with manipulation of guidewire and placement across the ductus. This is manifested by sudden deterioration in oxygenation as one is preparing the balloon-stent. If the guidewire is well positioned, it is a matter of rapidly going through the steps of stent implantation. However, if this occurs only at the stage of guidewire manipulation into the ductus, it is best to remove the wire and re-start the PGE1 infusion, recommencing the procedure after a period of stabilization. If the problem recurs, it is best to abandon the procedure and send the patient to surgery. Given its very low occurrence, it is not possible to predict which patient is likely to develop this complication. It is advisable to have the PGE1 infusion on standby in the catheterization laboratory.

### (C) Migration of expanded stent

This is not a life-threatening complication, but nonetheless serious as the patient needs to go to surgery semi-electively for stent removal and construction of a BT shunt. This is likely to occur if the pulmonary end of the ductus is not sufficiently constricted (>2.5 mm). It is advisable not to proceed with catheterization and leave the patient without PGE1 infusion for a longer period if the patient is only mildly cyanosed and has evidence of significantly large ductal flow clinically and on echocardiography. Once an expanded stent has migrated to the pulmonary artery, it is not possible to remove it except surgically. (The rates of serious complications quoted above are estimates based on our own single-center experience as the body of literature on the subject is still very small).

## POST-PROCEDURE CARE AND FOLLOW-UP

**Figure 8:**
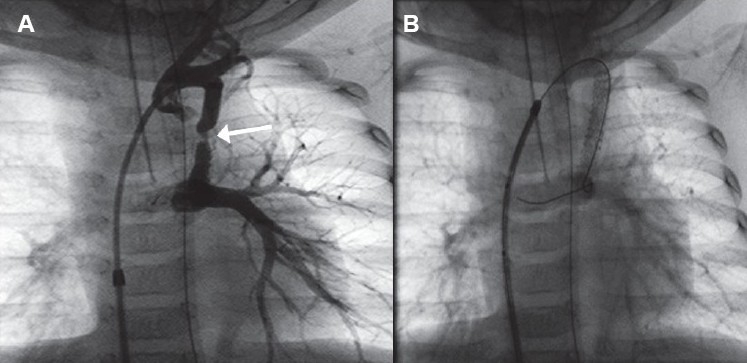
(A) Long tubular ductus from the left subclavian artery. Only the distal half of the ductus was stented. Two months postductal stenting. Severe stenosis of unstented ductus adjacent to the stent (arrow). (B) Second stent implanted to cover the entire length of the ductus

If stable hemodynamically, the patient is transferred to the ICU and kept ventilated for at least 12 h with arterial and central venous lines. It is common to have high, near-normal oxygen saturations in the first 48-72 h, but this is often well tolerated. Clinically severe heart failure from overshunting is uncommon and the patient should be extubated as early as possible. Heparin at 25 units/kg/h should be continued for 72 h and aspirin may be started the next day (if this has not already been started). Discharge and follow-up review are as per patients following creation of BT shunt. The durability of palliation by ductal stenting is generally less compared to that of surgical shunt; hence, a close follow-up is required and the definitive surgery should be planned within 6-18 months of ductal stenting. Re-crossing the stented ductus at follow-up catheterization, especially where a long stent was implanted and significant in stent stenosis has occurred, may be difficult. It is important to stent the entire length of the ductus as much as possible. There is a tendency for the unstented part of the ductus to become stenosed rapidly [Figure 8].

## CONCLUSION

Ductal stenting is a reasonable, less invasive alternative to BT shunt as first-stage palliation in neonates with duct-dependant cyanotic CHD. Crucial to the success of the procedure and medium-term outcome is case selection and choice of technique. This is largely based on the angiographic morphology of the ductus - its origin from the aorta, shape, length and size, tortuosity and its insertion onto the pulmonary artery.
